# Complete mitochondrial genome of the fish leech *Zeylanicobdella arugamensis*

**DOI:** 10.1080/23802359.2017.1372699

**Published:** 2018-05-31

**Authors:** Yongbo Wang, Min Huang, Rongxia Wang, Lanyu Fu

**Affiliations:** Research Institute of Marine Fisheries, Hainan Academy of Ocean and Fisheries Sciences, Haikou, P. R. China

**Keywords:** Mitochondrial genome, *Zeylanicobdella arugamensis*

## Abstract

The complete mitochondrial genome of the fish leech *Zeylanicobdella arugamensis* from China has been determined for the first time in this study. It was 16,161 bp in length and consisted of 22 tRNA genes, two rRNA genes, 13 protein-coding genes (PCGs), and one control region. The nucleotide base content of *Z. arugamensis* mitogenome was 35.5% A, 10.4% C, 10.4% G, and 43.7% T. Start codon ATG was used in PCGs, while most of the termination codons are incomplete T or TA. The tRNA genes were ranged from 59 bp (tRNA-Arg and tRNA-Glu) to 69 bp (tRNA-Gln and tRNA-Cys) in length. The phylogenetic tree was constructed and suggested that *Z. arugamensis* has closer relationship to the *Poecilobdella manillensis*, *Erpobdella octoculata*, *Hirudo hipponia*, *Whitmania acranulata*, *Whitmania pigra*, *Whitmania Laevis*, *Hirudo verbaba*, and *Hirudo medicinalis*, and that they constitute a sister group.

Parasitic infestation is one of the major problem for Asian sea bass cultured in cages (Leong et al. 2006). Marine leeches *Zeylanicobdella arugamensis* was considered to be the most common parasites in cultured marine fish. The first report of *Z. arugamensis* was from tank-reared grouper *Epinephelus coioides* in the Philippines (De Silva 1963), and later infections by *Z. arugamensis* have been reported in Sri Lanka, Malaysia, Singapore, India and Japan (De Silva and Fernando 1965; Sanjeeva et al. 1977; Cruz-Lacierda et al. 2000; Nagasawa and Uyeno 2009). In this study, the complete mitochondrial genome of the fish leech *Z. arugamensis* from China was sequenced for the first time, aiming to contribute to the species identification and phylogenetic study. The specimen was obtained from the grouper *Epinephelus fuscoguttatus* reared in Qionghai research base of Hainan Academy of Ocean and Fisheries Sciences (E110.68, N19.37). The specimen of *Z. arugamensis* was stored in Qionghai research Base and preserved in 95% ethanol.

The whole mitochondrial genome of *Z. arugamensis* is 16,161 bp in length (GenBank Accession number KY474378). It consists of 22 tRNA genes, two rRNA genes, 13 protein-coding genes (PCGs), and one control region. The nucleotide base content of *Z. arugamensis* mitogenome was 35.5% A, 10.4% C, 10.4% G, and 43.7% T. The 79.2% of (A + T) showed great preference to AT.

The mitochondrial genome of *Z. arugamensis* contains 22 tRNA genes varying from 59 bp to 69 bp in length. The 12s rRNA is 738 bp in length and located between tRNA-Met and tRNA-Val, and the 16s rRNA is 1155 bp in length, located between tRNA-Val and tRNA-Leu1. The mtDNA of *Z. arugamensis* consists of 13 PCGs, and all these genes begin with a standard ATG start codon. Most of the termination codons are incomplete T or TA, except for *CYTP*, *ND4L*, and *ND4*, which use TAA as stop codon. The control region is 1670 bp in length located between tRNA-Arg and tRNA-His.

The phylogenetic trees were construction based on the mitochondrial genome sequence of 14 fish leech (Clitellata: Hirudinea) species by Maximum Likelihood (ML) method. The result (Figure 1) has suggested that *Z. arugamensis* has closer relationship to the *Poecilobdella manillensis*, *Erpobdella octoculata*, *Hirudo hipponia*, *Whitmania acranulata*, *Whitmania pigra*, *Whitmania Laevis*, *Hirudo verbaba*, and *Hirudo medicinalis*, and that they constitute a sister group.

**Figure 1.**
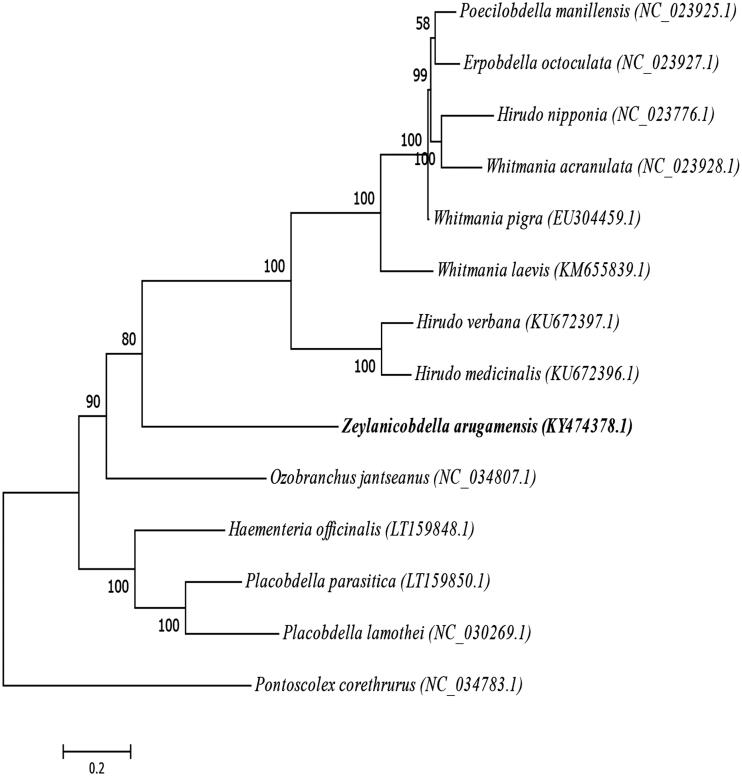
Phylogenetic tree derived from *Z. arugamensis* and the other leech’ mtDNA genome nucleotide sequences.
